# QTc interval prolongation during favipiravir therapy in an Ebolavirus-infected patient

**DOI:** 10.1371/journal.pntd.0006034

**Published:** 2017-12-28

**Authors:** Pierangelo Chinello, Nicola Petrosillo, Silvia Pittalis, Gianluigi Biava, Giuseppe Ippolito, Emanuele Nicastri, on behalf of the INMI Ebola Team

**Affiliations:** Lazzaro Spallanzani National Institute for Infectious Diseases (INMI), IRCCS, Rome, Italy; University of Geneva Hospitals, SWITZERLAND

## Introduction

Life-threatening arrhytmias, including *torsades de pointes* and ventricular fibrillation, may be induced by corrected QT (QTc) interval prolongation. Several antimicrobial drugs have been associated with QTc interval prolongation [1,2]. Favipiravir is an inhibitor of the RNA-dependent RNA polymerase of many RNA viruses, including influenza viruses, arenaviruses, phleboviruses, hantaviruses, flaviviruses, enteroviruses, and noroviruses [3]. Favipiravir has also been used in the recent epidemic of Ebolavirus (EBOV) in West Africa [4]. To date, no significant effects of favipiravir on the QT/QTc interval have been detected [5]. We report a case of QTc interval prolongation during favipiravir therapy in an EBOV-infected patient treated at our institution.

## Presentation of case

**Fig 1:**
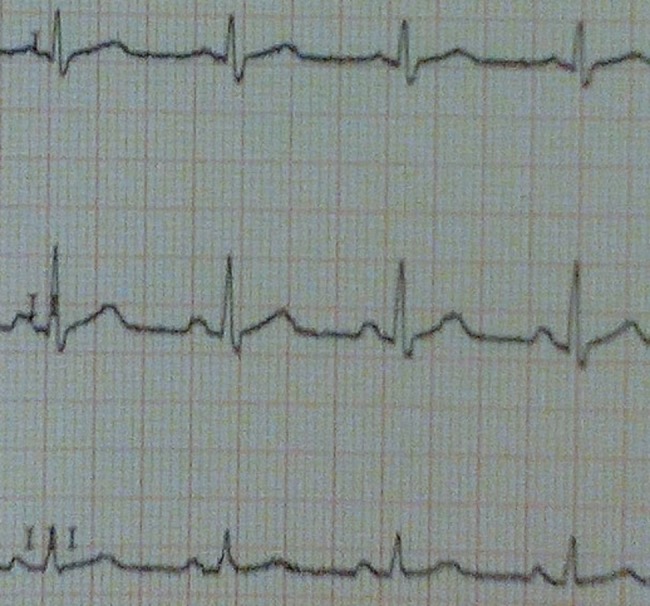
ECG registered on May 15, 2015.

**Fig 2:**
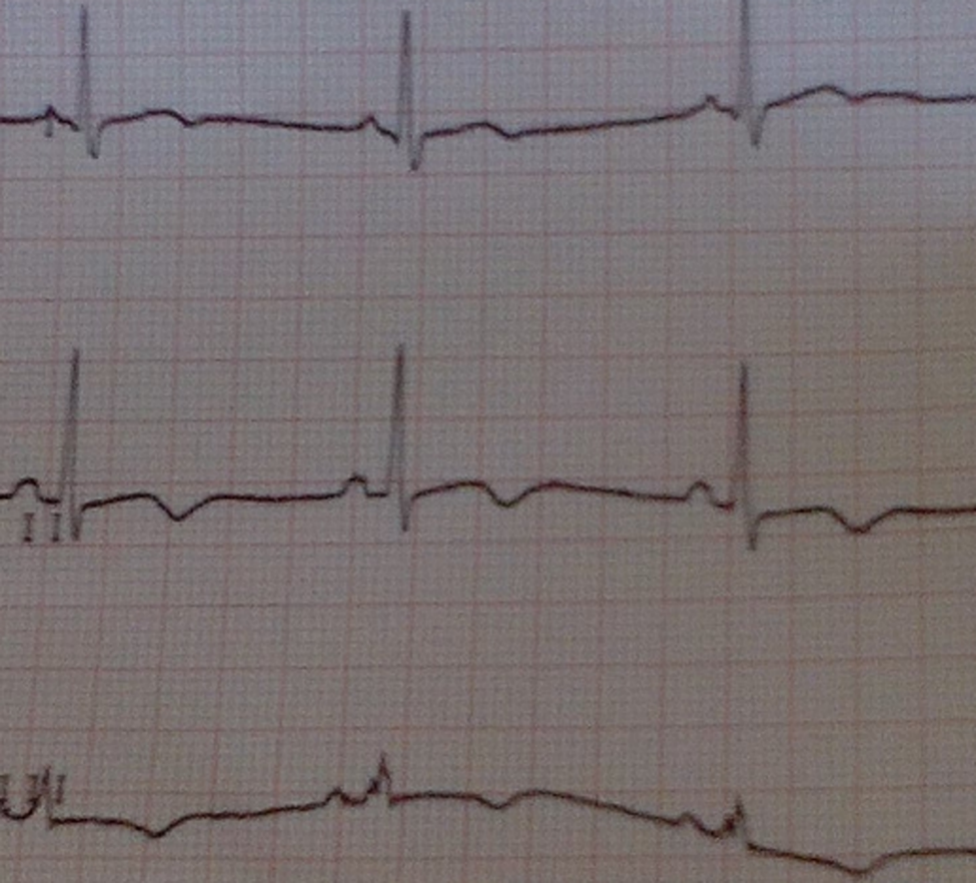
ECG registered on May 22, 2015.

**Fig 3:**
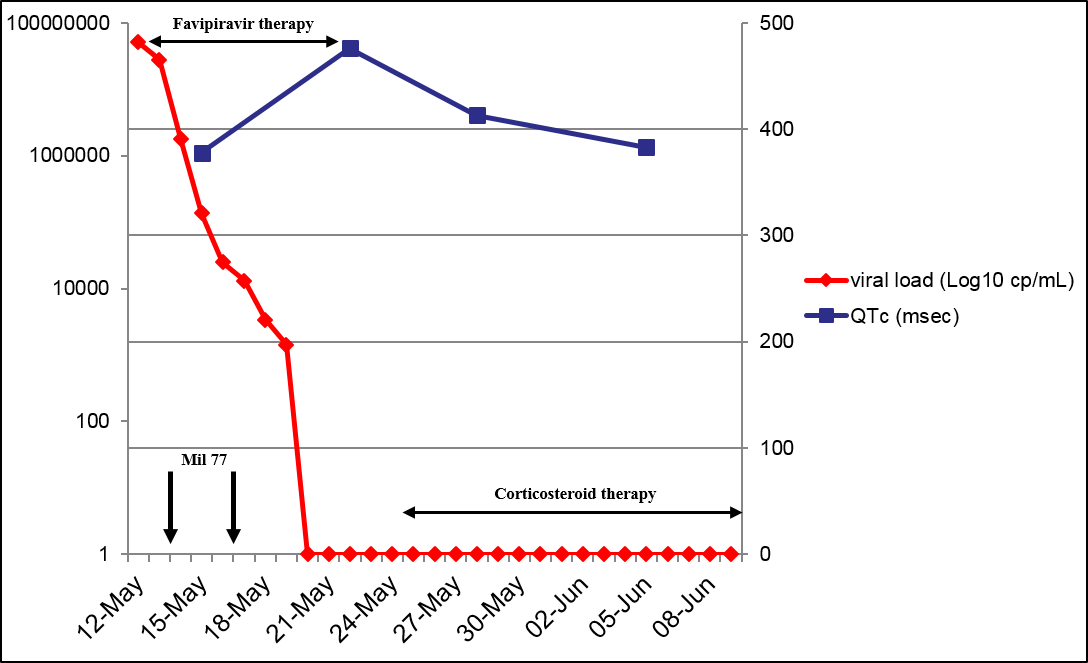
EBOV viral load and QTc interval over time. EBOV, Ebolavirus; QTc, corrected QT.

A 37-year-old Italian male nurse worked in Sierra Leone from February 15, 2015, to May 7, 2015, during the West Africa EBOV epidemic and subsequently returned home to Sardinia (Italy). A few days after his return, on May 10, 2015, he started complaining of fever, chills, and arthromyalgia. On May 11, he was admitted to the Sassari Hospital isolation unit, and his blood samples were sent to the Virologic Laboratory of the Lazarro Spallanzani National Institute for Infectious Diseases (INMI) in Rome, where the diagnosis of EBOV infection was made. On May 13, he was medically evacuated to the Lazzaro Spallanzani institute in high-isolation condition. On admission, his blood tests showed a leucocyte count of 4,000 cells/mmc (neutrophils 67%, lymphocytes 27%), haemoglobin 17.2 g/dL, platelets 79,000/mmc, glucose 77 mg/dL, creatinine 0.92 mg/dL, K^+^ 3.5 mEq/L, Na^+^ 147 mEq/L, aspartate aminotransferase 181 U/L, alanine aminotransferase 43 U/L, total bilirubin <0.5 mg/dL, and creatine phosphokinase 785 U/L; EBOV viraemia was 5 x 10^7^ cp/mL. He was febrile, prostrated, with mild dyspnea (oxygen saturation level [SatO_2_] 88% in room air), slow ideation, and diarrhoea. On May 15, a 12-lead ECG was recorded (Fig 1): the QT interval was 320 msec, the frequency rate was 84 bpm, and the QTc was 378 msec, as calculated by Bazett formula [6]. The patient was treated with oral favipiravir (Toyama Chemical Co., Ltd., Japan) from May 13 to May 22 (6 g on the first day and 1.2 g twice daily on the following days), mefloquine 250 mg 1 tablet/week (on May 8, 15, 23, and 30), furosemide 25 mg twice daily from May 14 to June 5, omeprazole 20 mg twice daily from May 14 to June 10, levofloxacin 750 mg daily from May 13 to 19, ceftriaxone 2 g daily from May 13 to 20, and intravenous rehydration; he also received two doses of investigational monoclonal antibodies against EBOV (MIL77; Mabworks, Beijing, China) on May 13 and 16. On May 22, the last day of favipiravir therapy, a QT interval of 480 msec, a pulse rate of 59 bpm, and a calculated QTc of 476 msec were recorded (Fig 2). On that day, plasma K^+^ was 3.95 mEq/L, Na^+^ 134 mEq/L, Ca^++^ 1.08 mmol/L, and creatine phosphokinase 31 U/L; no other cardiac biomarkers have been collected. After favipiravir withdrawal, the QTc interval decreased to 405, 413, and 383 msec on May 25, May 28, and June 5, respectively. Plasma EBOV viraemia was negative from May 21 (Fig 3). After an initial improvement of clinical conditions with disappearance of fever, diarrhoea, and dyspnea, the clinical course was complicated by a new onset of fever on May 24 associated with lymphadenopathy, petechial skin rash, and low platelet count. Pericardial effusion was detected by echocardiography on May 25; no further evidence of pericarditis, myopericarditis, and/or ischemia has been found. No signs of new infections were detected, and in the suspicion of an immunologic overreaction to EBOV infection, the patient was given steroid treatment starting May 26 with progressive normalisation of the clinical picture. He was discharged from the hospital in good general conditions and with a QTc interval within normal limits on June 10, 2015.

## Case discussion

Favipiravir is a pyrazinecarboxamide derivative released in 2002 in Japan as an inhibitor of influenza virus replication [7]. It subsequently proved activity against several classes of viruses, including EBOV [8], and was used in both therapy and postexposure prophylaxis during the recent EBOV epidemic in West Africa [9]. In their recent work, Kumagai and colleagues [5] found no effect of favipiravir on the QT interval in healthy Japanese adults after administration of single oral doses of favipiravir 1,200 and 2,400 mg. To the best of our knowledge, there are no other studies assessing this issue [4,10]. Our patient was treated with favipiravir doses much higher than those reported by Kumagai and colleagues, i.e., 6 g on day 1 and 2.4 g on the following days, and this could explain a previously unreported effect on QTc interval prolongation. Other drugs administered to the patient, namely levofloxacin and mefloquine, had the potential to prolong the QTc interval. Levofloxacin was withdrawn three days before the registration of the longest QTc interval, and it is known to have an elimination half-life of 6.0 hours to 8.9 hours after oral and intravenous doses in patients with normal renal function [11]; however, tissue accumulation of levofloxacin could have had a role in increasing the patient’s susceptibility to drugs with QTc-prolonging potential. On the other hand, mefloquine exhibits a considerably high cardiac safety index [12], and its administration as antimalarial prophylaxis continued until May 30, 2015, when QTc interval was already shortened after favipiravir withdrawal, thus suggesting that it had a minor role in this patient’s QTc interval prolongation. A prolonged use of proton pump inhibitors (PPIs) has been associated with hypomagnesaemia and subsequent QT interval prolongation [13]. We could not dose Mg plasma levels in our patient, but the short use of PPI and the reduction of QTc interval while continuing PPI therapy make the role of omeprazole unlikely in prolonging the QTc interval. Other electrolyte disturbances, particularly hypokalaemia, may induce QTc interval prolongation. Our patient, however, had K^+^, Na^+^, and Ca^++^ within the normal range when the longest QTc interval was recorded.

QTc prolongation has already been described in other EBOV-infected patients treated outside Africa [14,15]. In the first case, it was attributed to metabolic acidosis in the context of severe sepsis [14]. Chertow et al. attributed the QTc elongation to concomitant administration of propofol, but their patient presented with myocarditis—confirmed by imaging—that could have also contributed to the QTc prolongation [15]. Evidence of pericarditis has also been described at autopsy in a macaque who experienced a delayed death with a decline in clinical condition after first apparent clinical recovery [16], and presence of EBOV antigen is well described within cardiac tissue and pericardial bag [17].

Whether the development of a cardiac effusion and its etiology could have impacted the QTc in our patient remains unclear. The prolonged QTc is detected only 3 days before the cardiac effusion has been noted. Unfortunately, we did not perform any cardiac imaging on May 22, 2015, when the QTc interval prolongation was noted. Pericardial effusion may have been already present at that time, and we cannot exclude that correction of the QTc could have been influenced also by the steroid therapy that resolved the effusion and the immunologic reaction. However, the ECGs performed on May 23, May 24, and May 25, (before the beginning of steroid therapy) showed a QTc of 453, 476, and 405 msec, respectively: the normalisation of the QTc interval before the beginning of steroid therapy, together with the short plasma half-life of favipiravir [18], suggests a more likely role of favipiravir withdrawal in QTc interval normalisation.

Encephalitis or central nervous system (CNS) pathology may have a role in prolonging QTc interval. However, the patient’s slow ideation noted on admission improved quickly, and no signs of encephalitis or CNS pathology were present when the QTc interval prolongation was recorded.

In conclusion, we suggest that favipiravir administered at high doses, together with the cofactors discussed above, may have contributed to inducing a QTc interval prolongation in our EBOV patient. Early extinguishing of levofloxacin and removal of the favipiravir were sufficient for correction over time.

The QTc interval reached 476 msec on the last day of favipiravir therapy; the side effect was therefore mild and should be weighed against the benefits expected in the treatment of one of the most dreadful infections in the world. If feasible, ECG monitoring could be advisable during high-dose favipiravir therapy, especially when patients experience electrolyte disturbances and concomitant use of drugs with QTc-prolonging potential.

## Ethics statement

The patient gave consent to have his case details published. The authors’ institutional ethics committee approved this study.

**Key learning points**

- In the treatment of EBOV infection, favipiravir is used at higher doses than in the treatment of influenza.
- High doses of favipiravir could cause side effects unrecognised at common doses.
- Electrolyte disturbances are common during EBOV infection and may induce QTc interval prolongation.
- ECG monitoring could be advisable during high-dose favipiravir therapy, especially when patients experience electrolyte disturbances and concomitant use of drugs with QTc-prolonging potential.
